# Physical activity and self-esteem in domestic and foreign medical students

**DOI:** 10.1192/j.eurpsy.2024.1412

**Published:** 2024-08-27

**Authors:** E. Nikolaev, A. Zakharova, G. Dulina, D. Hartfelder, S. Petunova, N. Grigorieva, E. Lazareva, N. Maksimova, E. Litvinova, E. Vasilieva, S. S. Fakhraei

**Affiliations:** ^1^Department of Psychiatry, Medical Psychology and Neurology; ^2^Department of Social and Clinical Psychology; ^3^Medical Faculty, Ulianov Chuvash State University, Cheboksary, Russian Federation

## Abstract

**Introduction:**

It is a well-known fact that regular physical activity (PA) has a positive effect on a person’s somatic health. Does PA have similar correlations with self-esteem in medical students of different cultural backgrounds?

**Objectives:**

To determine the intensity and correlations of PА and self-esteem in domestic and foreign undergraduate medical students

**Methods:**

We carried out a survey of 305 domestic and 241 international medical students of both genders at Ulianov Chuvash State University. For this aim we used the Sociocultural Health Questionnaire (E. Nikolaev)

**Results:**

We have established that with the same duration of the sessions the average frequency of physical activity (PA) of foreign medical students is higher than that of domestic students (p=.001). The latter more often exercise in gyms (p=.001) and consume bodybuilding supplements (p=.01). Foreign medical students’ self-assessment of their health (p=.001) and sportiness (p=.001) is higher than that of domestic students (7.90 vs 6.98 и 6.72 vs 5.82 correspondingly). Higher frequency of PA correlates in domestic medical students with higher self-assessment of their successfulness (r=.47), attractiveness (r=.46), and confidence (r=.43); while in foreign students – of their sportiness (r=.49), confidence (r=.25), sociability (r=.23). Longer sessions of PA by domestic medical students are interrelated with higher self-assessment of their intellect (r=.35), confidence (r=.34), happiness (r=.34); while in foreign students – of sportiness (r=.47), health (r=.36), and successfulness (r=.36).

**Conclusions:**

The revealed data testify to the fact that PA of both domestic and foreign medical students closely correlates with positive assessment of their own personality.

**Disclosure of Interest:**

None Declared

